# Initiation of gametocytogenesis at very low parasite density in Plasmodium falciparum infection

**DOI:** 10.1093/infdis/jix035

**Published:** 2017-05-10

**Authors:** Ryan Farid, Matthew W. Dixon, Leann Tilley, James S McCarthy

**Affiliations:** 1 QIMR Berghofer Medical Research Institute and University of Queensland, Brisbane, Australia; and; 2 Department of Biochemistry and Molecular Biology, Bio21 Molecular Science and Biotechnology Institute, University of Melbourne, Victoria, Australia

**Keywords:** *Plasmodium*, Gametocyte EXported Protein-5, early gametocyte marker, experimentally induced blood stage malaria, piperaquine.

## Abstract

The recent focus on the elimination of malaria has led to an increased interest in the role of sexual stages in its transmission. We introduce *Plasmodium falciparum* gametocyte exported protein-5 (PfGEXP5) transcript analysis as an important tool for evaluating the earliest (ring) stage sexual gametocytes in the blood of infected individuals. We show that gametocyte rings are detected in the peripheral blood immediately following establishment of asexual infections—without the need for triggers such as high-density asexual parasitemia or drug treatment. Committed gametocytes are refractory to the commonly used drug piperaquine, and mature gametocytes reappear in the bloodstream 10 days after the initial appearance of gametocyte rings. A further wave of commitment is observed following recrudescent asexual parasitemia, and these gametocytes are again refractory to piperaquine treatment. This work has implications for monitoring gametocyte and transmission dynamics and responses to drug treatment.

*Plasmodium falciparum* causes >200 million cases of malaria each year and kills approximately 400000 children [1]. Treatment of malaria is heavily reliant on a class of drugs called the artemisinins, delivered as a combination with a limited repertoire of partner drugs. Thus, it is extremely concerning that resistance to artemisinins and to all of the partner drugs is now evident in South East Asia, leading to increasing numbers of clinical failures (up to approximately 50% in some regions) [2, 3]. Resistance to the partner drug piperaquine is a particular emerging problem in Cambodia, underpinning lower cure rates [4–6].

As the World Health Organization (WHO) shifts its focus from disease control to elimination, it is critically important to understand the drivers and dynamics of carriage of both asexual parasites, which cause disease, and sexual-stage gametocytes, which are responsible for disease transmission. Gametocytogenesis is initiated when a sexually committed merozoite invades a red blood cell (RBC) [7]. The sexually committed parasite remains inside its host RBC but undergoes a remarkable morphological change as it transforms from a cell optimized for multiplication in the bloodstream of humans to a cell capable of undergoing sexual reproduction in a mosquito. The *P. falciparum* gametocyte transitions through 5 distinct stages over a period of about 10 days. Early ring stage gametocytes are morphologically indistinguishable from asexual rings. Later stage gametocytes (stages II–IV) of *P. falciparum* gradually elongate to adopt a characteristic crescent or falciform shape [8]. Stage II–IV gametocytes disappear from the circulation, apparently by sequestering in deep tissues, including the spleen and bone marrow [9]. The only morphologically recognizable gametocyte stage observed in the peripheral circulation in humans is stage V, which re-enters the peripheral circulation and becomes available for uptake by mosquitoes.

Given the lack of specific markers for ring-stage gametocytes, it has been difficult to answer a number of fundamental questions, such as whether these early stage gametocytes are sequestered or are freely circulating [9, 10]. Indeed, questions remain as to whether gametocyte commitment occurs in the bloodstream or in a privileged environment such as the bone marrow. Similarly, it is not clear whether gametocyte production is a constitutive event, with a subpopulation of parasites converting to sexual development during each asexual replication cycle, or an induced event, triggered by exposure to density-dependent changes in nutrient conditions or by environmental stresses, such as a host immune response [11–14]. Importantly, it has been suggested that exposure to certain drugs, including the 4-aminoquinolines, or to suboptimal drug treatment (as occurs during the emergence of drug resistance) with other drug classes, can promote sexual commitment [15, 16]. Without tools to study commitment in vivo (ie, validated and sensitive sexual ring stage markers), definitively answering these questions is very difficult.

Piperaquine is a bisquinoline antimalarial that was developed in the 1960s. It was first used as a monotherapy in China, leading to the development of resistance in that country [17]. It competes with chloroquine for uptake, inhibits β-hematin formation, and is active only against the mature stages of intraerythrocytic asexual parasites [18–20], leading to the general assumption that it exerts its antimalarial activity through the same mechanism as chloroquine. However, it shows little cross-resistance with chloroquine, indicating that it is not a substrate for extrusion from the digestive vacuole through the *P. falciparum* chloroquine resistance transporter (PfCRT) [20]; indeed emerging evidence suggests that resistance is mediated by amplification of the genes encoding hemoglobin-degrading enzymes plasmepsin 2 and 3 [21, 22]. Piperaquine is coformulated with dihydroartemisinin in a widely used artemisinin combination therapy. The emergence of resistance to both piperaquine and artemisinin in Cambodia has led to concerns that treatment with dihydroartemisinin/piperaquine combinations may enhance gametocyte carriage and promote the spread of resistance to both drugs.

The experimentally induced blood-stage malaria (IBSM) infection model [23] has proven very useful for monitoring the outcomes of different treatments and for informing deployment of those drugs. For example, this model was used to show that monotherapy of *P. falciparum* infections with piperaquine rapidly clears asexual parasitemia but is followed some time later by the appearance of mature gametocytes [24]. However, it was not clear from this study whether these gametocytes were induced by drug treatment or were formed prior to treatment.

*Plasmodium falciparum* gametocyte exported protein-5 (PfGEXP5) has been recently identified as a specific marker of early stage sexually differentiated parasites [25]. In this article, we report that measuring *gexp5* transcript levels provides a convenient and reliable method for enumerating ring-stage gametocytes both in vitro and in vivo. Using quantitative reverse transcriptase assays specific for *gexp5* (gametocyte rings), skeleton-binding protein-1 (*sbp1*; asexual and gametocyte rings), and *P. falciparum* sexual-stage antigen-25 (*pfs25*; late-stage gametocytes), we have characterized the production of gametocytes in experimental human malaria infections before and after treatment with piperaquine monotherapy.

## METHODS

### In Vitro Parasite Culture

Very tightly synchronized schizont stages (3% parasitemia, 5% hematocrit) of a high gametocyte-producing 3D7 parasite line [26] were obtained as previously described [27] and allowed to reinvade at high density. Samples were harvested during the first cycle after reinvasion, N-acetyl-glucosamine was added to remove asexual stage parasites [28], and further samples were collected as gametocytogenesis progressed.

### Clinical Study Design

The clinical study component (ANZCTR no. 12613000565741) was conducted at the contract research organization, Q-Pharm (Brisbane, Australia). Blood samples were collected from subjects in the third cohort of a previously reported study whose objective was to investigate the antimalarial activity of piperaquine [24]. The PCR data that were used to measure parasitemia by 18S rDNA qPCR from the previously published study was reused in the study reported here.

### Polymerase Chain Reaction Analysis

Parasitemia in the clinical trial was monitored using a real-time, quantitative PCR using primers and a hydrolysis probe that targets the 18S rDNA gene [29]. For quantification of specific parasite gene transcripts, primer pairs and hydrolysis probes were designed to amplify the following transcripts: *gexp5, sbp1, pfs25,* and *18S* ribosomal RNA, using assays described in detail in the Supplementary Materials. The assays were designed to exclude amplification of any contaminating genomic DNA targets. Sample mRNA and controls were reverse-transcribed to cDNA, and qRT-PCR was performed as described (Supplementary Materials). Positive, negative, and “no template” controls were included, whereas standard curve material (7 × 10-fold dilutions) was comprised of linearized and purified plasmid DNA target standards. These standard curves were used to report the absolute number of mRNA transcripts in samples by interpolation using a linear regression model of the measured concentration (transcripts per microliter) of the standard curve material (Supplementary Material).

## RESULTS

### *gexp5* Transcript as a Marker for Ring-Stage Gametocytes

**Figure 1.**
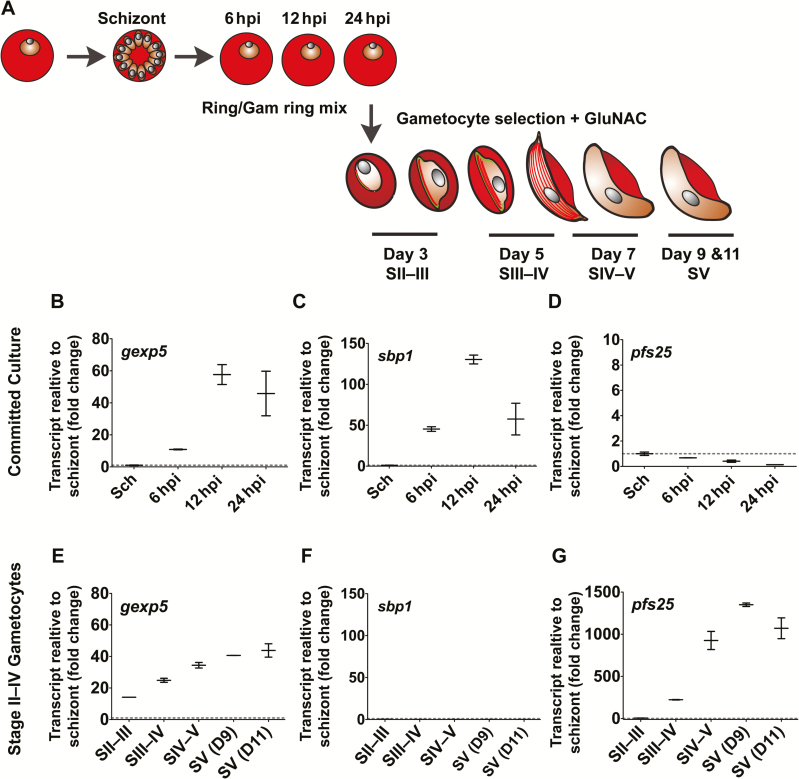
*A*, Schematic representation of the in vitro commitment experiment. Tightly synchronized 3D7 strain parasites (0–2 hours post infection [hpi]) were used to initiate a culture at 3% parasitemia. Parasites were harvested at schizont (Sch) stage (cycle 1, 42 hpi), and at 6, 12, and 24 hpi. The culture was treated with N-acetyl-glucosamine (GluNAC) to remove asexual stage parasites, and samples were harvested on days 3 (stage II–III [SII–III]), 5 (stage III–IV [SIII–IV]), 7 (stage IV–V [SIV–V), 9 (early stage V [SV]), and 11 (late stage V). The transcript abundance of *gexp5* (*B, E*), *sbp1* (*C, F*), and *Pfs25* (*D, G*) was determined at each of these time points. All values shown are normalized to 18S rRNA and expressed as fold-change over the schizont control sample. The mean values and 5–95 percentile error bars are shown.

Gametocyte exported protein-5 (GEXP5, PF3D7_0936600) can be detected in the ring stage of parasites that are committed to sexual-stage development from 14 hours post invasion (hpi) but is absent in asexual rings [25]. To determine the transcriptional profile of *gexp5*, tightly synchronized (approximately 2-hour window) schizonts (approximately 3% parasitemia) were allowed to reinvade, forming a high parasitemia culture (approximately 13%) containing a proportion of committed gametocytes. Two hours after reinvasion, any remaining schizonts were removed, and samples of the culture were harvested at 6, 12, and 24 hpi (Figure 1A, top row).

*gexp5* is transcribed at only very low levels in schizont-stage parasites (Figure 1B). The transcript level (normalized to the 18S rRNA signal) increases significantly by 6 hours after reinvasion at high density. The transcript level peaks at 12 hpi, with a 55-fold increase compared with schizont-stage parasites, and remains high at 24 hpi. (Figure 1B). A somewhat similar pattern of transcription is observed for the skeleton-binding protein 1 (SBP1, PF3D7_0501300); however, the level of this transcript declines more dramatically at 24 hpi (Figure 1C). As expected, levels of the late-stage (female-specific) gametocyte marker, *pfs25* (PF10_0303), are low in schizonts and during the early stages of commitment, with residual signal likely representing low-level contamination with late-stage gametocytes (Figure 1D).

We subjected the 24-hpi culture to treatment with N-acetyl-glucosamine to remove asexual parasites and examined transcript levels in cultures containing sexual parasites from stage II to stage V of development (Figure 1E–G). Levels of the *gexp5* transcript appear to increase steadily from stage II to stage V of development (Figure 1E); however, it should be pointed out that transcript levels are normalized to the 18S rRNA signal. Thus, they may appear artificially low in the early stages of gametocyte development due to the presence of nonviable but still present asexual parasites. Nevertheless, the level of gexp5 transcripts was sufficient in parasites beyond Stage I to preclude measurement of this transcript in isolation as a marker the exclusive presence of early stage gametocytes. In contrast, the *sbp1* transcript remains very low across stage II to stage V of sexual development (Figure 1F). These data demonstrate the usefulness of the *gexp5* transcript as a marker for the presence of early-stage gametocytes in mixed populations of asexual and sexual parasites, as well as across the entire developmental phase. By contrast, *sbp1*, which is expressed in both asexual and sexual-stage rings [30], is not useful in differentiating asexual parasites from early-stage gametocytes in mixed populations. As expected, we see a dramatic rise in *pfs25* transcript levels from stage IV of development, reaching >1000-fold increase in stage V compared with the schizont stage (Figure 1G). These data indicate that measurement of all 3 of these markers simultaneously will provide a means of determining which lifecycle stages are present in the circulation.

### Onset of Gametocytogenesis in the Bloodstream From the First Round of Asexual Replication

**Figure 2.**
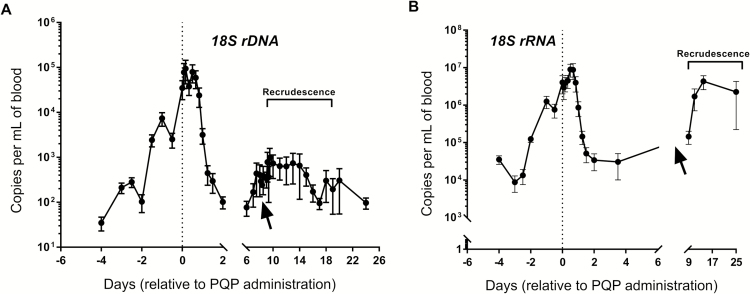
*A*, 18S genome copies (numbers of parasites) in the peripheral blood of subjects before and after piperaquine treatment. Day 0 = first treatment with piperaquine (PQP). *B,* Abundance of 18S rRNA (MAL_18S:rRNA) transcript, as measured using 2-step quantitative reverse transcriptase PCR method (qRT-PCR). Levels were interpolated by linear regression analysis using cloned plasmid DNA calibration curves. Dotted lines indicate time of administration of a single 480-mg dose of piperaquine. Arrows indicate timing of a second dose of piperaquine (960 mg) for all except for 1 patient, who received the second dose 1 day earlier. n = 6. Standard error of the mean is shown. Negative (no detection) PCR results are given a value of 1. Data were reanalyzed from a previous study [24].

We used these markers to analyze samples collected from a subset of subjects who had participated in a previously reported IBSM study [24]. Six malaria-naive, healthy male and nonpregnant female subjects aged 18–50 years were infected with the reference strain of *P. falciparum*, 3D7. A typical growth pattern of asexual parasitemia was observed in all study subjects (Figure 2A), with parasitemia becoming detectable by qPCR (18S rDNA) on day 4 after infection (4 days before treatment). The number of parasite genomes increased in the log sinusoidal pattern that is characteristic of a synchronous infection, with peak periods of circulating rings stages followed by periods of sequestration of mature stage-infected RBCs, then a further increase following schizont rupture. A similar trend was evident when qRT-PCR was undertaken to quantify 18S rRNA transcripts using a 2-step qRT-PCR method (Figure 2B). Subjects were treated with 480 mg of piperaquine on day 8 after infection, upon reaching the predetermined threshold of >1000 parasites/mL, which led to a dramatic decrease in the 18S rDNA and 18S rRNA signals 1 day after treatment. Three subjects showed recrudescent parasitemia on days 11, 23, and 27 after infection, when they received a second larger dose of piperaquine (960 mg; arrows). Recrudescence can be identified as a resurgence in the levels of parasite 18S DNA and rRNA (Figure 2A and 2B, right side).

**Figure 3.**
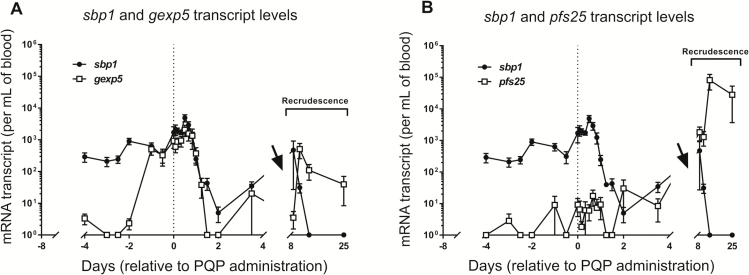
Abundance of mRNA transcript in the peripheral blood of subjects, as measured using 2-step quantitative reverse transcriptase PCR (qRT-PCR). Levels were interpolated by linear regression analysis using cloned plasmid DNA calibration curves. The thin dotted lines indicate time of administration of a single 480-mg dose of piperaquine. Arrows indicate timing of a second dose of piperaquine (960 mg) for all except for 1 patient, who received their second dose 1 day earlier. n = 6. Standard error of the mean is shown. Negative (no detection) qRT-PCR results are given a value of 1. *A, gexp5* and *sbp1*. *B, pfs25* and *sbp1*.

We measured levels of *gexp5*, *sbp1,* and *pfs25* mRNA transcripts in the peripheral blood of the subjects to obtain a more detailed view of the temporal course of transcription of these mRNA markers of asexual and sexual parasites. By day 4 after infection (4 days before treatment), *sbp1* transcript levels were readily detectable, consistent with the presence of circulating ring-stage parasites (Figure 3A). Levels of *sbp1* transcript increased on day 5, then exhibited peaks and troughs, synchronous with those observed for18S rRNA and rDNA, as the infection progressed. Again, this is consistent with cycles of asexual replication.

*gexp5* transcription was measurable from day 4 of the infection (Figure 3A), indicating that committed rings are present in the peripheral circulation in *P. falciparum*–infected humans. The production of gametocyte rings appears to occur very early in the blood-stage infection, when the parasite density is very low (approximately 100 per milliliter) and well before antimalarial treatment with piperaquine was administered. A trough in *gexp5* transcript levels on days 5–6 is consistent with disappearance of the first round of ring-stage gametocytes due to sequestration. A marked increase in *gexp5* transcript levels was observed on day 7 (Figure 3A), 2 days after the increase in the level of *sbp1* and *18S* rRNA transcripts on day 5. This is consistent with an increase in the rate of gametocyte production accompanying the rise in asexual parasitemia.

### Failure of low dose piperaquine to clear gametocytemia

Following piperaquine treatment on day 8 of the induced infection, the *18S* rRNA, *sbp1* and *gexp5* mRNA transcripts continued to rise for another 24 hours (Figures 2 and 3). This is consistent with the known poor activity of piperaquine against ring-stage parasites [19]. From day 9, both *sbp1* and *gexp5* transcript levels plummeted, consistent with killing of mature asexual parasites and ablation of the next cycle of asexual and sexual ring-stage parasites (and potentially the sequestration of the maturing gametocytes). A rebound in *sbp1* transcript level, accompanied by an increase in *gexp5* transcript, was observed in the 3 subjects who experienced recrudescent infection on days 11, 23, and 27 after infection (Figure 3A, right side). Levels of these transcripts decreased again after a second dose of piperaquine was administered.

The late-stage gametocyte marker *pfs25* is expected to be present in the circulation of *P. falciparum*–infected subjects only once mature (stage V) gametocytes appear in the circulation [31]. Accordingly, levels of *pfs25* mRNA transcripts remained low both before and after piperaquine treatment (Figure 3B). Consistent with the proposed time course of gametocytogenesis and the presence of this transcript only in mature gametocytes, the level of *pfs25* transcript increased dramatically on day 16 of the infection (Figure 3B, right side). This observation is in agreement with the release of mature gametocytes in the circulation 9 days after the first surge of *gexp5*-expressing rings on day 7 of the infection. Levels of *pfs25* transcripts continued to increase despite further treatment with piperaquine, consistent with limited sensitivity of developing gametocytes to this drug.

## DISCUSSION

In this article, we provide evidence that *gexp5* is transcribed from 6 hpi in ring-stage sexually committed parasites. Transcription of *gexp5* continues throughout gametocyte maturation, making it a useful marker across the entire sexual development phase. Although a transcriptional signal cluster associated with early ring-stage gametocytes has been reported previously[10], the identification of a single probe that detects ring-stage gametocytes and one that can be detected in vivo provides a convenient tool that can be used to investigate very early events in *P. falciparum* sexual differentiation. The fact that these transcripts appear and disappear from the blood of subjects with induced malaria infection, in a pattern consistent with the hypothesized timing of development of gametocytes, highlights the utility of this marker for in vivo studies.

The process of gametocytogenesis is of significant interest because it underpins transmission of malaria. For example, there has been considerable interest in the question of whether a fixed proportion of asexual schizonts commits to gametocytogenesis at every round of replication or whether gametocytogenesis is triggered by environmental cues. During in vitro culture, sexual conversion rates can be increased by increasing the asexual parasitemia [32] or otherwise stressing the culture [33], suggesting that density-dependent factors control the decision made at this developmental branch point. However, another study [14] showed that the expression profile of a gametocyte-specific gene, *pfge1*, correlated well with asexual parasitemia, leading to the suggestion that gametocytogenesis occurs in a constitutive fashion during in vitro culture.

In this study, we followed asexual and sexual blood-stage development in subjects, using *gexp5* as a gametocyte-specific ring marker and *sbp1* as a marker of all rings. The *sbp1* transcript was detected 4 days after induced blood-stage infection, at the same time point at which 18S rRNA and rDNA were first detected. The *gexp5* transcripts were detected at a low level on the same day. This is consistent with the initiation of sexual commitment at the earliest stages of the erythrocytic lifecycle, when the parasite density is <100 parasites per milliliter (approximately 0.000002% of RBCs infected). In this context, it should be noted that the limit of detection with Giemsa thick film is approximately 10000 parasites per milliliter [34].

Interestingly we found that the level of *gexp5* transcript increased dramatically as the parasitemia rose. The paucity of quantitative data prevents us from concluding whether this is more consistent with density-dependent enhancement of commitment or constitutive commitment that rises with increasing parasitemia. Nevertheless these data indicate that gametocytogenesis occurs at very low densities and in clinical malaria is very likely already at a high level by the time patients develop symptoms. These presymptomatic individuals will potentially contribute to malaria transmission.

There has been significant interest in determining the compartment in which commitment takes place. Stage II–IV gametocytes are not observed in the peripheral blood due to sequestration in the spleen and bone marrow [9]. Gametocytes do not appear to express surface adhesins capable of binding to endothelial cells [35] in the same manner as asexual parasites, and it has been suggested that sequestration is driven by the altered mechanical properties of the developing gametocytes [28, 36, 37]. If this model is correct, ring-stage gametocytes would be present in the circulation. An alternative hypothesis is that committed merozoites enter the hematopoietic compartment of the bone marrow and invade RBC precursors and then remain and develop in this compartment [38]. Experimental testing of these alternative hypotheses in humans has been difficult until now due to a lack of suitable tools. Here we demonstrate that ring-stage gametocytes are indeed present in the peripheral circulation in human infections, providing strong evidence that this is the compartment in which commitment can take place, a conclusion that is supported by analyses of transcriptional cluster profiles associated with natural infections in patients [10].

We followed changes in circulating asexual and sexual ring stages after treatment with piperaquine. The number of asexual and sexual parasites continued to increase following treatment, consistent with a previous study showing that piperaquine has little activity against ring-stage parasites [19]. After 24 hours, the circulating ring-stage parasites declined rapidly, consistent with killing of mature asexual parasites, thus preventing further rounds of gametocyte production. Mature (*pfs25*-positive) gametocytes appeared in the circulation 9–10 days after the appearance of the initial surge of ring-stage (*gexp5*-positive) gametocytes and continued to circulate even after the follow-up treatment with a higher dose of piperaquine. This indicates that sequestered gametocytes are not killed by the piperaquine exposure achieved in this study. This is consistent with the relatively poor activity of piperaquine in vitro against midstage gametocytes [39, 40].

The parasite line used in this study, 3D7, is piperaquine sensitive; however, there is increasing evidence for the emergence of resistance to this drug in the Greater Mekong subregion. Moreover, dose selection for piperaquine is complicated by variable levels of absorption [41], by different pharmacokinetic profiles in different patient groups [42, 43], and by potential side-effects at high doses [44]. There is evidence that some patients are underdosed at the standard adult regimen of 400 mg per day for 3 days, and current dosing in young children is considered suboptimal [45]. This underdosing will be further exacerbated as drug resistance spreads. It should also be kept in mind that 3D7 had been laboratory adapted prior to its use in these experimental infection studies, and therefore replication of these findings with field isolates would be desirable.

This study reveals that gametocytogenesis is initiated very early in human infections, almost certainly prior to the point at which patients seek treatment. Coupled with the lack of activity of piperaquine against both asexual and sexual ring-stage parasites and its inadequate activity against mid- and late-stage gametocytes, this raises significant concerns about using combinations that include piperaquine, particularly in the face of emerging resistance. The observation that DHA-piperaquine is associated with higher levels of gametocytemia after treatment, compared with other artemisinin combinations [46–48], merits highlighting. As piperaquine is assessed as a partner in new combinations with OZ439 [48] and KAE609 [49], it will be important to consider the ability of potential partner drugs to prevent transmission. The ability of gametocytes to survive piperaquine exposure would be expected to contribute to the transmission of parasites with reduced drug sensitivity. This may have contributed to the appearance of piperaquine resistance within a few years of its introduction into the field in China [50] and more recently in the Greater Mekong subregion [6, 51].

Efforts to eradicate malaria from particular regions will be highly dependent on effective methods of targeting gametocytes. The IBSM model offers a very useful tool to determine whether individual components and particular drug combinations are likely to carry the liability of permitting gametocyte production. Moreover, the availability of a *gexp5* transcript-based assay with sufficient sensitivity to detect ring-stage gametocytes when the parasite density is <100 parasites per milliliter provides a very important tool to address key epidemiological questions relating to the dynamics of parasite transmission. These tools are critical to current efforts to progress toward malaria elimination.

## Supplementary Material

Supplementary Data

## Notes

***Acknowledgments.*** J. M. C., R. F. M. W. D and L. T. designed the experiments. R. F. and M. W. D executed the experiments and analyses. All authors undertook data analysis and drafted and reviewed the manuscript. The authors would like to acknowledge the clinical trial volunteers and the staff from Q-Pharm in the conduct of the clinical trial that generated the samples used in this work, including Dr Paul Griffin, Dr Suzanne Elliott, and Gem Macenroth. The authors would also like to thank Dr Silvana Sekuloski and Greg Robinson from QIMR Berghofer for assistance in design of the study and project management, and technical laboratory assistance, respectively. The authors thank Joerg Moehrle, MMV, and Pietro Alano, Istituto Superiore di Sanità, Italy, for helpful discussions.

***Financial support.*** This work was supported by the National Health & Medical Research Council of Australia (1098992, 1041802, 1037304), the Australia Research Council (FL150100106), Medicines for Malaria Venture, and the Bill and Melinda Gates Foundation.

***Potential conflicts of interest.*** All authors: No reported conflicts. All authors have submitted the ICMJE Form for Disclosure of Potential Conflicts of Interest. Conflicts that the editors consider relevant to the content of the manuscript have been disclosed.
