# Supplementary material for: Initiation of gametocytogenesis at very low parasite density in Plasmodium falciparum infection
Source: J Infect Dis. 2017 May 10;215(7):1167–74. doi: 10.1093/infdis/jix035 (PMC5426372; doi:10.1093/infdis/jix035)
Supplement: Supplementary Data [file jix035_Supplementary_Data.zip › 2017_01_06_Supplemental_Material_GEXP5_McCarthy_Rev1_Clean.docx]

**SUPPLEMENTAL MATERIAL**

**J Infectious Diseases**

**Gametocytogenesis is initiated at very low parasite densities in malaria infections and is refractory to piperaquine**

Ryan Farid^1^, Matthew W. Dixon^2^, Leann Tilley^2^, James McCarthy^1^

^1^QIMR Berghofer Medical Research Institute and University of Queensland, Brisbane, Australia;

^2^Department of Biochemistry and Molecular Biology, Bio21 Molecular Science and Biotechnology Institute, The University of Melbourne, Melbourne, Victoria, Australia

**Supplemental Methods:**

**1. Primer and probe sequences**

**2. RNA extraction**

**3. cDNA synthesis**

**4. RT-PCR assays**

**1. Primer and probe sequences**

| **ASSAY TARGET** | **ASCESSION NO. (PLASMODB)** | **PRIMER AND PROBE SEQUENCES**  **(5’ – 3’)** | | **HYDROLYSIS PROBE FLUOROPHORE** | **AMPLICON SIZE (*bp*)** | **SOURCE Ref** |
| --- | --- | --- | --- | --- | --- | --- |
| ***gexp5*** | PF3D7_0936600 | FORWARD:  REVERSE:  HYDROLYSIS PROBE: | CTTCTTGTTCGAGATTATCCCT  GGAGTCTACTAATTCAGACAGC  TGTAATGTAGTAGAAGGTACCATTGGTCA | HEX™  [ZEN™/IB®FQ]^b^ | 110 | - |
| ***pfs25*** | PF3D7_1031000 | FORWARD:  REVERSE:  HYDROLYSIS PROBE: | AAATCCCGTTTCATACGCTTGTAA  CAGTTTTAACAGGATTGCTTGTATCTAATATAC  ACCAAATGAATGTAAGAATGTAACTTGTGGTAACGGT | FAM™  [BHQ®-1] | 80 | [^1^](#_ENREF_1) |
| ***18S rRNA*** | PF3D7_0531600 | FORWARD:  REVERSE:  HYDROLYSIS PROBE: | AGGAAGTTTAAGGCAACAACAGGT  GCAATAATCTATCCCCATCACGA  TGTCCTTAGATGAACTAGGCTGCACGCG | Cy5™  [IB®RQ] | 152 | [^2^](#_ENREF_2) |
| ***sbp1*** | PF3D7_0501300 | FORWARD:  REVERSE:  HYDROLYSIS PROBE: | GCAAAACAAGCCGTACATGTTG  TTGCTAGGTAATATCCTTTTCTTTTTCC  TTGTTCATCAACTTTTACAACTT | FAM™  [NFQMGB]^a^ | 80 | [^3^](#_ENREF_3) |

^a^ *‘MGB’ probe (Applied Biosystems)*

^b^ *Double-Quenched Probe (Integrated DNA Technologies)*

**2. RNA extraction**

Preserved parasitized RBCs were thawed and centrifuged (10,000 × g, 10 min) at 4°C. Supernatants were immediately aspirated and total RNA extraction was carried out using the Ambion™ PureLink® RNA Mini Kit (Thermo Fisher Scientific) with some minor modifications made to the recommended vacuum blood protocol. 800 µL of the preserved patient blood samples (containing 133 µL parasitized RBC) were used. Capped Spin-Away™ filters (Zymo Research, Cat. #C1006) were used to reduce potential genomic DNA (gDNA) contamination. This gDNA removal step followed homogenization with lysis buffer (β-mercaptoethanol added), but before adding ethanol to the samples, and was carried out by centrifugation (8000×g, 1 min). The optional on-column DNase digestion step was used (PureLink® DNase Set, Invitrogen), but with 100 µL used per column. Upon eluting the purified total RNA with 40 µL of nuclease-free water, the eluent was divided into ~15 µL aliquots in 0.1 mL tubes (Eppendorf). Purified RNA samples were analysed using both Nanodrop 2000c and Qubit® RNA HS Assay Kit (Invitrogen) to assess RNA yield and quality.

**3. cDNA synthesis**

Sample RNA and controls were reverse transcribed to cDNA using the PrimeScript™ RT Reagent Kit with gDNA Eraser (Takara Clontech). A set of ‘no RT control’ (-RT) samples were also made to confirm the absence of gDNA-derived PCR signal. Other controls include a positive, negative and ‘no template control’ (nuclease-free water), 20 U (1U/µL) recombinant RNase Inhibitor
(Takara Clontech) replaced the removed RT enzyme in the -RT mastermix. The maximum RNA template volume, 7 µL of the 20 µL cDNA sample final volume, was used for all samples and controls. The manufacturer’s recommended protocol was followed without variation. Following cDNA synthesis, all samples (including –RT samples) were diluted 1/1.75 by adding 15 µL of nuclease-free water and stored at -20°C before being analysed.

**4. RT-PCR assays**

***Validation of RT-qPCR Assays***

Assays were validated for performance when multiplexed equivalent to how they performed in singleplex (data not shown). Additionally, Ct value reproducibility was confirmed by using positive and negative controls, as well as other clinical samples. Reproducibility of all PCR assays was undertaken by determining the % Coefficient of Variation on technical replicates of PCR reactions run in triplicate (i.e. to ensure the replicate Ct values were close [SD/mean x 100, where SD is the standard deviation]). CV values of ~5% or less were observed for all targets between PCR runs (data not shown).

***Controls Samples***

In-vitro cultured 3D7 Plasmodium *falciparum* of 3-7% parasitemia (mixed-stage) were used as positive controls in the RT-qPCR assays. As a negative control, bloods collected from malaria-naïve human volunteers were used.

***RT-qPCR Mastermix Concentrations and Cycling Conditions***

The RT-PCR mastermix used was QuantiNova Probe PCR Kit (QIAGEN). The product instructions were followed except the final MgCl_2_ concentration was 3mM. Primers and probe concentrations were 100nM each and 50nM respectively. 1.5 µL of diluted cDNA template or standard were added to the wells with 3 technical replicates. The total reaction volume used was 15 µL per well. The PCR cycled 35 times: 5 s at 4°C, 30 s at 60°C. Hard-Shell® 384-Well 480 Plates (Bio-Rad) were used for the PCR reactions using a CFX384 (Bio-Rad) instrument.

***Standard curve material***

To generate standard curve material for quantitative analysis, the target PCR products were cloned into a plasmid vector, and plasmid DNA template produced in bulk by *in vitro* culture, and linearised by digestion with Restriction enzyme NotI (New England BioLabs, #R0189). Double-stranded linearised plasmid DNA (ds LpDNA) was purified by column chromatography and then quantitated by Qubit dsDNA HS assay kit (Thermo Fisher Scientific, #Q32851).

| **ASSAY TARGET**  **ds LpDNA** | **CONC.**  **[%CV]** | **LENGTH** | **MOLECULAR MASS ^#^** | **ds LpDNA Copies/µL** | **ds LpDNA STANDARD**  **MAXIMUM CONC. (Copies/well)^^^** |
| --- | --- | --- | --- | --- | --- |
| ***gexp5*** | 7.06 ng/µL  [3.46] | 3157 bp | 3.459 × 10^-9^ ng | 2.041 × 10^9^ | 3 × 10^6^ |
| ***pfs25*** | 2.04 ng/µL  [5.85] | 3161 bp | 3.464 × 10^-9^ ng | 5.893 × 10^8^ | 3 × 10^6^ |
| ***18S rRNA*** | 4.98 ng/µL  [5.52] | 3220 bp | 3.455 × 10^-9^ ng | 1.411 × 10^9^ | 3 × 10^7^ |
| ***sbp1*** | 9.49 ng/µL  [1.08] | 3153 bp | 3.459 × 10^-9^ ng | 2.746 × 10^9^ | 3 × 10^6^ |

*^#^ Molecular Mass (ng) = ds LpDNA Length* $\times\left( \frac{660}{6.023 \times{10}^{23}} \right)\times{1000}^{3}$

*^^^ 1.5 µL template cDNA and Standards used per PCR well*

***PCR performance***

| **ASSAY TARGET** | **PCR EFFICIENCY**  **(*%*)** | **R^2^** | **LIMIT OF QUANTIFICATION**  **(transcripts per µL pDNA)** | **STANDARD CURVE CV (*%*)** |
| --- | --- | --- | --- | --- |
| ***gexp5***  Plasmodium exported protein (PHISTc) | 100.2 | 0.9947 | 18 | 0.78 |
| ***pfs25***  25kDa ookinete surface antigen precursor | 102.3 | 0.9992 | 80 | 0.04 |
| ***18S rRNA***  18S ribosomal RNA | 96.4 | 0.9994 | 152 | 0.06 |
| ***sbp1* *(ring stage-specific marker)***  Skeleton-binding protein 1 | 99.4 | 0.9985 | 80 | 0.23 |

The dilution range for the standards for the different target genes covered 7 orders of magnitude (10-fold dilutions). The diluent was EASY Dilution Solution (Takara Clontech). The Table above outlines further details of the LpDNA standards for each assay. Diluent-only blanks were also included.

**References**

1. Pasay CJ, Rockett R, Sekuloski S, et al. Piperaquine monotherapy of drug-susceptible *Plasmodium falciparum* infection results in rapid clearance of parasitemia but is followed by the appearance of gametocytemia. *J Infect Dis*. 2016;214(1):105-113.

2. Rockett RJ, Tozer SJ, Peatey C, et al. A real-time, quantitative PCR method using hydrolysis probes for the monitoring of Plasmodium falciparum load in experimentally infected human volunteers. *Malar J*. 2011;10:48.

3. Joice R, Nilsson SK, Montgomery J, et al. *Plasmodium falciparum* transmission stages accumulate in the human bone marrow. *Sci Transl Med*. 2014;6(244):244re245.
